# Drug-Coated Balloon vs. Drug-Eluting Stents for De Novo Unprotected Left Main Stem Disease: The SPARTAN-LMS Study

**DOI:** 10.3390/jcdd10020084

**Published:** 2023-02-16

**Authors:** Tharusha D. Gunawardena, Natasha Corballis, Ioannis Merinopoulos, Upul Wickramarachchi, Johannes Reinhold, Clint Maart, Sulfi Sreekumar, Chris Sawh, Trevor Wistow, Toomas Sarev, Alisdair Ryding, Tim J. Gilbert, Allan Clark, Vassilios S. Vassiliou, Simon Eccleshall

**Affiliations:** 1 Department of Cardiology, Norfolk and Norwich University Hospital, Norwich NR4 7UY, UK; 2 Norwich Medical School, University of East Anglia, Norwich NR4 7TJ, UK; 3 Royal Brompton Hospital, London SW3 6NP, UK

**Keywords:** drug-coated balloon, left main stem, drug-eluting stent, complex coronary intervention

## Abstract

The objective of this study is to compare the outcomes of patients treated with drug-coated balloons (DCBs) or second-generation drug-eluting stents (DESs) for de novo unprotected left main stem (LMS) disease. Previous studies comparing the treatment of LMS disease suggest that the mortality for DES PCI is not worse than CABG. There are limited data from studies investigating the treatment of de novo LMS disease with DCB angioplasty. We compared the all-cause and cardiac mortality of patients treated with paclitaxel DCB to those with second-generation DES for de novo LMS disease from July 2014 to November 2019. Data were analysed using Kaplan–Meier analyses and propensity-matched analyses. A total of 148 patients were treated with either a DCB or DES strategy. There was no significant difference in all-cause mortality in the DCB group (19.5%) compared to the DES group (15.9%) (HR 1.42 [0.61–3.32], *p* = 0.42). Regarding cardiac mortality, 2 (4.9%) were recorded for the DCB group and 7 (6.5%) for the DES group (HR 1.21 [0.31–4.67], *p =* 0.786); for target vessel myocardial infarction, there were 0 (0%) for the DCB group and 7 (6.5%) for the DES group; and for target lesion revascularisation, there were 3 (7.3%) in the DCB group and 9 (8.3%) in the DES group (HR: 0.89 [0.24–3.30]). *p* = 0.86. These remained not significant after propensity score matching. We found no difference in the mortality outcomes with DCB angioplasty compared to second-generation DES, with a median follow-up of 33 months. DCB can therefore be regarded as a safe option in the treatment of LMS disease in suitable patients.

## 1. Introduction

Coronary artery disease affecting the left main stem (LMS) is associated with a high risk of morbidity and mortality if left untreated [1]. Traditionally, coronary artery bypass grafting (CABG) was the mainstay of treatment in patients requiring revascularisation for LMS disease and percutaneous coronary intervention (PCI), reserved only as a “salvage” option [2,3,4]. With increasing evidence supporting the use of PCI [2,5,6,7], this is now a recognised and guideline-indicated alternative approach [8,9,10].

Drug-eluting stents (DESs) have been associated with a number of pitfalls since their inception, including stent thrombosis [11,12], neo-atheroma [13], prolonged mandated dual antiplatelet therapy [14,15], and impaired vasoreactivity causing endothelial dysfunction [16,17]. Drug-coated balloons (DCBs) are a guideline-recommended treatment for in-stent restenosis (ISR), with a growing amount of data demonstrating their long-term efficacy in small-vessel de novo disease [18,19,20]. It can potentially reduce the strategic complexity of treating important LMS bifurcation, which can otherwise necessitate intricate stenting approaches [21], whilst mitigating the concerning risk of stent thrombosis in this scenario and promoting reduced sheer stress and natural vasomotion [22,23]. To date, there is only scarce evidence of DCB utilisation in LMS disease, limited mainly to ISR LMS lesions [12,24].

In this study, we aimed to compare the outcomes of patients treated with DCB or second-generation DES for de novo LMS disease.

## 2. Materials and Methods

The Safety of PAclitaxel dRug-coaTed balloon-only Angioplasty of de novo Left Main Stem coronary disease (SPARTAN LMS) study is an investigator initiated, single-centre cohort study. In our institution, PCI data are collected prospectively in a clinical database as described previously [19]. Institutional approval for this work was obtained from the Norfolk and Norwich Hospital with ethical approval from Northwest Heydock Research Ethical Committee and the ethics committee of the University of East Anglia. The Confidentiality Advisory Group within the Health Research Authority waived the need for patient consent for this retrospective study. Clinical and angiographic details were obtained from our clinical database. All clinical outcomes were obtained from a national database of clinical outcomes, Hospital Episodes Statistics. This provided ICD-10 diagnostic codes, which were used to identify patient outcomes, ensuring that all hospital admissions, including those outside of our centre, were reported. We retrospectively interrogated the database specifically looking for LMS coronary intervention with DCBs or DES. In order to ensure homogeneity, this analysis included patients with a primary lesion treated in the LMS rather than in the ostial left anterior descending (LAD), ostial ramus intermediate (RI), or ostial circumflex (Cx) arteries. The first DCB de novo LMS intervention was undertaken in July 2014, and we included all subsequent, consecutive patients who received either a DCB or DES for de novo LMS disease from July 2014 until November 2019.

Patients were excluded if they had received a combination of a DCB and a DES, or if they had received BMS. Three patients who were treated with both a DCB and a DES were excluded.

PCI of the LMS due to iatrogenic catheter-induced dissections were excluded due to conceivable pathological differences in disease. Patients who were in cardiac arrest, ventilated, or in cardiogenic shock were excluded. LMS protection was defined as the presence of a patent left internal mammary artery (LIMA) or a saphenous vein graft (SVG) to the left anterior descending (LAD) artery or the circumflex artery, and they were excluded from analyses.

All coronary angiograms were reviewed by two experienced blinded operators to classify bifurcational disease according to Medina bifurcation and ABCD LMS classification scores [25,26]. “True” bifurcation lesions were defined as having significant (>50%) stenosis in both the main branch (MB) and the side branch (SB) (i.e., Medina 1,1,1; 1,0,1; or 0,1,1), whereas all other lesion types were classified as “non true.” Lesion length was obtained using the length of the DES or the DCB, or the sum if more than one device was used. The vessel diameter was obtained from the maximum diameter of the DCB or DES used, or the post-dilatation balloon. This is a visual estimation in keeping with routine clinical practice and using a 1:1 balloon/stent to vessel ratio based on guideline recommendations [9,10,27]. The primary endpoint was all-cause mortality and the secondary endpoints were cardiovascular mortality, re-infarction (myocardial infarction of the target vessel), and target lesion revascularisation (which is defined as per the Academic Research Consortium [28]). Survival information was obtained from the UK Health and Social Care Information service (a UK national body where all deaths in the UK are recorded by law), and death certificates were obtained from the General Register Office in the UK. Causes of death were adjudicated by a committee blinded to the treatment strategies to identify cardiovascular vs. non-cardiovascular deaths according to the recent consensus paper on the classifications of cardiovascular outcomes [28].

Statistical analyses were carried out using STATA version 16.1 in which nominal variables were compared using Chi-squared tests, while continuous variables were compared using Student’s t-test. Kaplan–Meier plots were used to estimate survival. Statistical significance was defined as *p* < 0.05. Propensity score matched analysis was performed using a Cox proportional-hazard model.

As this is a retrospective analysis, all decision making in terms of treatment strategy was at the discretion of the operator, with patient and lesion characteristics taken into account. The use of DCB was only considered by operators with a large amount of DCB experience.

## 3. Results

**Figure 1:**
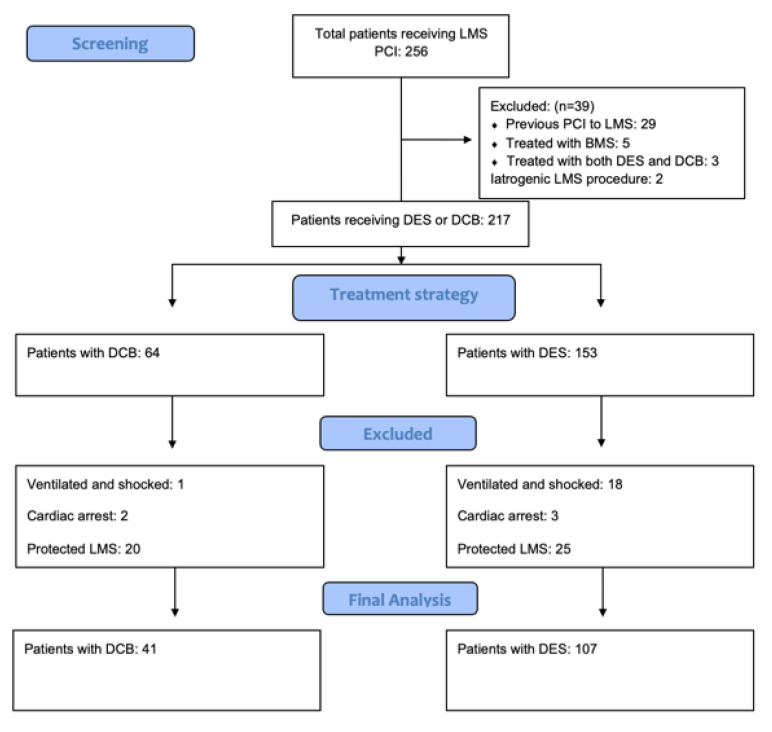
Study composition—a diagram to demonstrate the exclusions to form the final study cohort.

A total of 148 patients were identified as meeting the inclusion criteria. A total of 41 were treated with a DCB and 107 were treated with a DES. Figure 1 identifies the study composition and those excluded from the study. The median follow-up was 33.9 ± 20 months. Of the three patients treated with DCB and DES, two were treated with a bail-out stent after a persisting dissection with a DCB, and the third was a hybrid approach with a two-stent DES strategy and a DCB to a small intermediate vessel. There were no adverse events in these patients.

The 41 patients who were treated with a DCB were all treated with an iopromide paclitaxel DCB, which included 33 SeQuent Please NEO DCBs (80% of all the DCBs) and 8 SeQuent Please DCBs (20% of all the DCBs).

Of the 107 patients treated with a DES, 36 (34%) received the RESOLUTE ONYX Zotarolimus DES [28,29], 27 (25%) received the Promus Premier Everolimus DES, 27 (25%) received the SYNERGY Everolimus DES, 8 (7%) received the XIENCE Everolimus DES, 5 (5%) patients received the Promus Element Everolimus DES, and 4 (4%) received the Terumo ULTIMASTER Sirolimus DES.

There was a significant trend within the DCB group of older patients than those receiving DES (mean age 73.8 ± 11.7 versus 69.8 ± 11.1 *p =* 0.048). Table 1 illustrates the patient composition of the two groups. The DCB group was treated with a significantly shorter duration of dual antiplatelet therapy (*p* < 0.01).

The DCB group contained a significantly greater proportion of true bifurcations (73.2% vs. 48.6%, *p =* 0.006), suggesting that DCB was the preferred strategy for more challenging anatomical distributions. Furthermore, DCB procedures involved using less contrast (mean 144.5 ± 41.3 mL) compared with DES procedures (with mean 176.5 ± 67.1 mL/ *p =* 0.006). The stent procedures involved greater utilisation of intracoronary imaging, which was implemented in 35.9% of the DCB procedures versus 76.6% of the DES procedures (*p* < 0.001). The DCB group had a smaller average vessel diameter (3.8 ± 0.3 mm in the DCB group versus 4.3 ± 0.6 mm in the DES group, *p* < 0.001). Vessel diameter was calculated using the largest balloon used from either pre- or post-dilatation or the deployment balloons for the DES/DCB. Vessel length was the length of the DES/DCB (or summative if there were multiple DESs/DCBs used) and, on average, DCB-treated segments were non-significantly shorter (21.71 versus 24.72, *p* = 0.20). (Table 2). The presence of a coronary dissection after the delivery of a DCB was assessed in all patients, with a type A dissection in 9.8% and a type B dissection in 39.0% of lesions. Two lesions had a persistent unsafe dissection after delivery of a DCB, and these were treated with a DES as a bail out.

**Figure 2:**
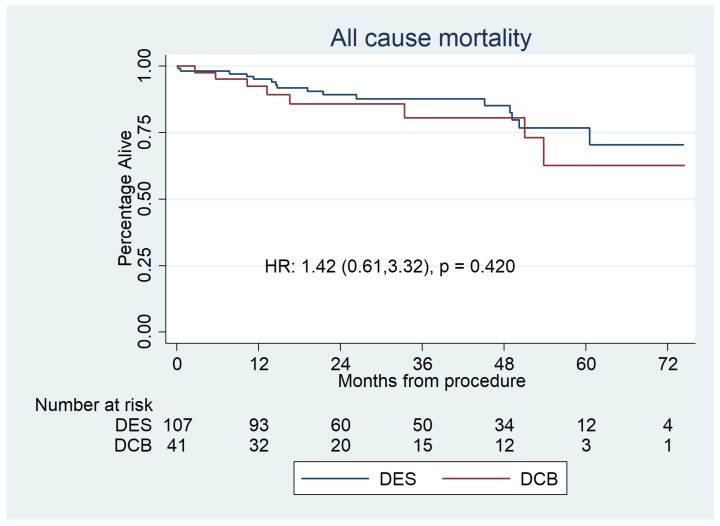
Kaplan–Meier estimator plot of all-cause mortality for paclitaxel DCBs vs. second-generation DESs for de novo LMS disease. The number at risk is represented at the bottom. This shows no significant difference between the groups.

All-cause mortality for the DCB group was 8 (20%), whereas it was 16 (15%) in the DES group (HR 1.42 [0.61–3.32], *p* = 0.42). Mortality was analysed using Kaplan–Meier estimator plots (Figure 2).

A Cox proportional-hazard model was fitted using propensity score weighting. The weights were estimated from a logistic regression using the covariates of age, gender, hypercholesterolaemia, hypertension, peripheral vascular disease (PVD), previous stroke/cerebrovascular accident (CVA), prior myocardial infarction (MI), previous PCI, prior CABG, heart failure, history of angina, asthma, COPD, diabetes, AF, eGFR, true bifurcational disease, and method of vascular access. For the proportional-hazards models, the assumptions were checked using Schoenfeld residuals.

**Figure 3:**
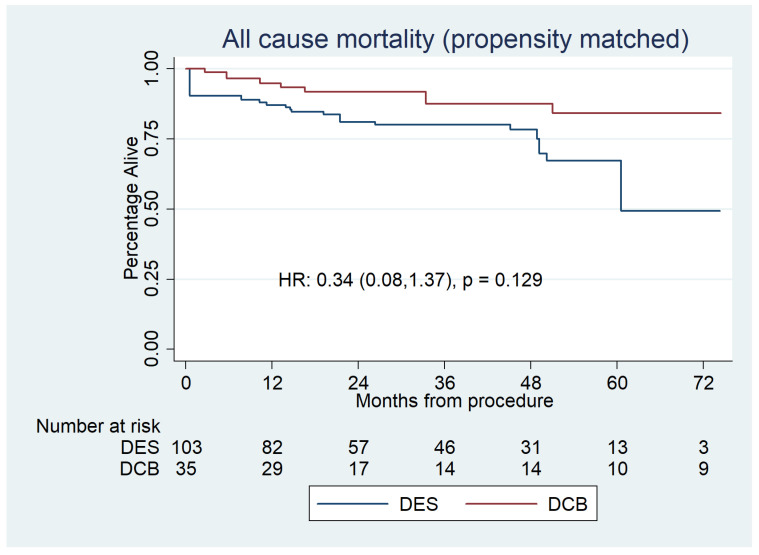
Propensity-matched Kaplan–Meier survival plot for all-cause mortality for DCBs versus DESs. This shows no significant difference between the groups.

The propensity analyses found no significant difference between DCBs and DESs in all-cause mortality (Figure 3).

**Figure 4:**
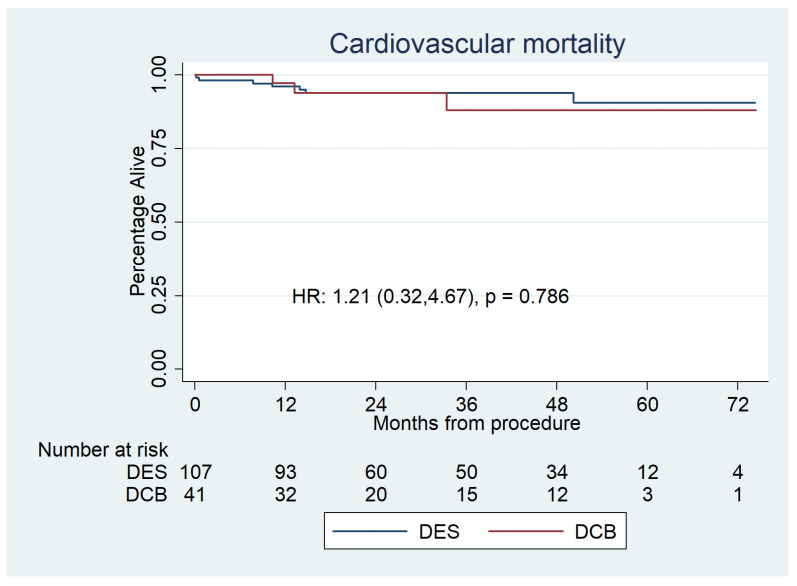
Kaplan–Meier estimator analysis of cardiovascular mortality for paclitaxel DCBs vs. second-generation DESs for de novo LMS disease. It shows no significant difference between the groups.

**Figure 5:**
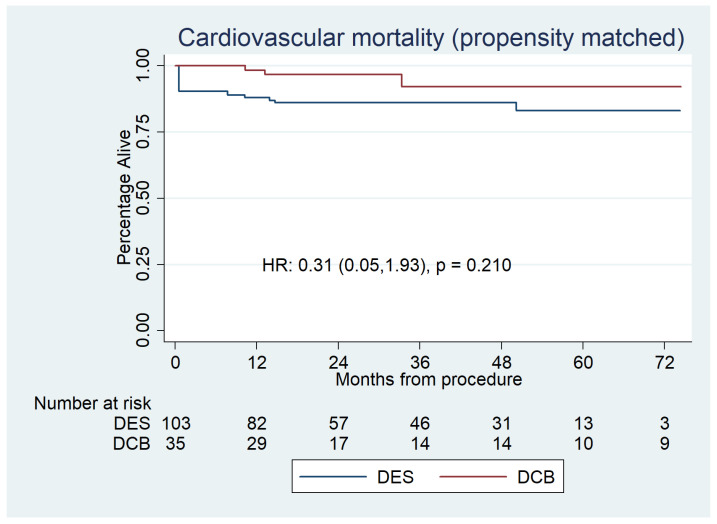
Propensity-matched Kaplan–Meier survival plot for cardiovascular mortality for DCBs versus DESs, which shows no significant difference between the groups.

Cardiovascular mortality was 2 (4.9%) for the DCB group and 7 (6.5%) for the DES group (HR 1.21 [0.31–4.67], *p =* 0.786) (Figure 4), which was also non-significant after propensity score matched analysis [HR:0.31 (0.05–1.930, *p* = 0.210] (Figure 5).

**Figure 6:**
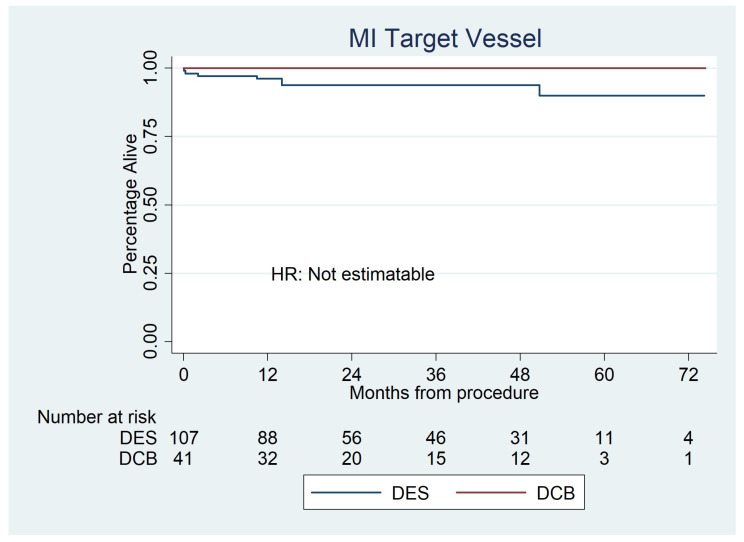
Kaplan–Meier estimator analysis of target vessel myocardial infarction for paclitaxel DCBs vs. second-generation DESs for de novo LMS disease. Due to the low event rate, it was not possible to calculate hazard ratios.

Target vessel myocardial infarction occurred in 0 (0%) DCBs and 7 (6.5%) DES as shown in Figure 6.

**Figure 7:**
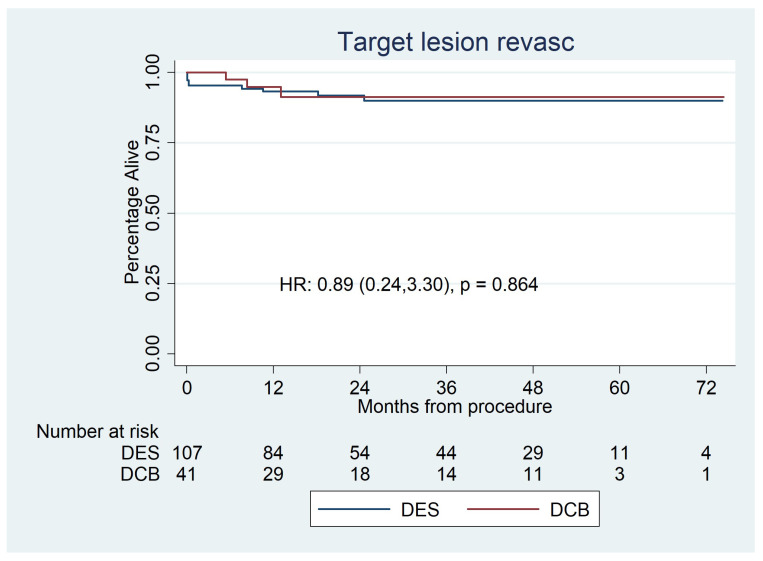
Kaplan–Meier estimator analysis of repeat revascularisation for paclitaxel DCBs vs. second-generation DESs for de novo LMS disease. It shows no significant difference between the groups.

There was no significant difference in the rates of target lesion revascularisation between the two groups, with 3 (7.3%) TLRs in the DCB group and 9 (8.3%) in the DES group (HR: 0.89 (0.24–3.30), *p* = 0.86) (Figure 7).

Propensity score matched analysis was not performed for target vessel MI or target lesion revascularisation in light of low event rates.

Of the lesions treated with a two-stent strategy, sub-group analysis was performed. This confirmed no difference between treatment strategies in all-cause mortality (HR: 0.83 (0.43–3.6), *p* = 0.7).

**Figure 8:**
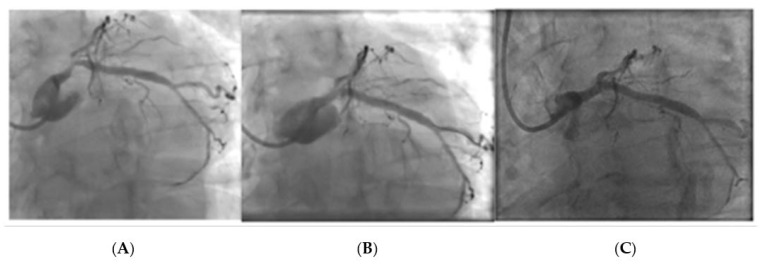
Left main stem PCI with DCB- Image (**A**): Pre PCI, Image (**B**): Immediately post-PCI, Image (**C**): 6-month follow-up angiogram.

**Figure 9:**
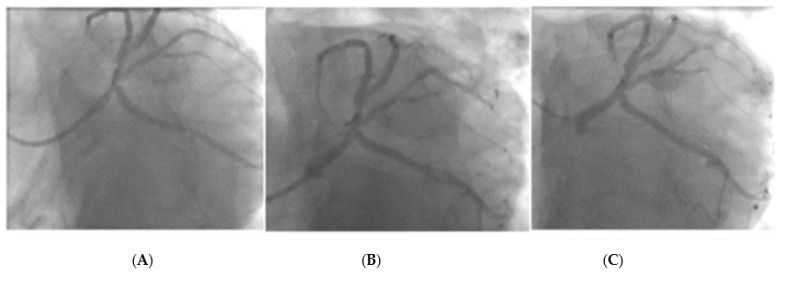
Left main stem DCB PCI with image (**A**): Pre PCI, Image (**B**): Immediately post PCI, Image (**C**): 6-month follow-up angiogram.

Figure 8 and Figure 9 represent two cases treated with a DCB with a follow-up angiography at 6 months. Both patients were considered as candidates for PCI after heart team discussion.

**Figure 10:**
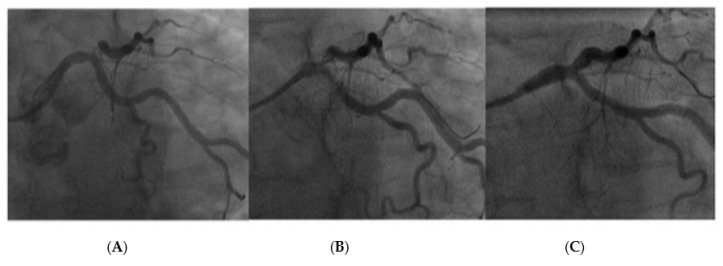
LMS lesion requiring bail-out stent due to dissection with ongoing chest pain after DCB and contrast hang-up. Image (**A**): LMS pre-treatment, Image (**B**): Dissection after delivery of DCB with persistent contrast hang-up and increasing lumen compromise, Image (**C**): Post-DES final result.

**Figure 11:**
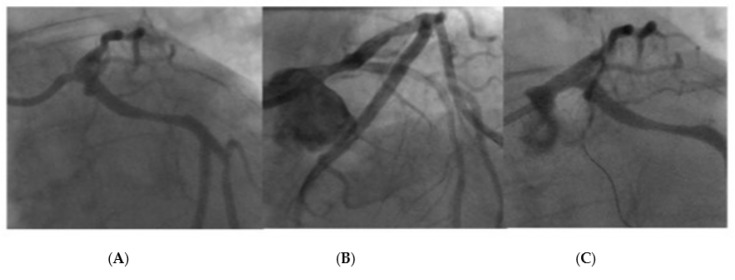
LMS lesion requiring bail-out stenting after DCB. This patient had an appearance of a type B dissection however there was persistent contrast hang-up in the dissection, resulting in bailout stenting. Image (**A**): Lesion pre-treatment, Image (**B**): Type B linear dissection after DCB, Image (**C**): Lesion after final DES treatment.

Figure 10 and Figure 11 represent two cases that required bail-out stenting after the delivery of a DCB. Case 1 was associated with a combination of a filling defect, vessel recoil, and ongoing chest pain, resulting in the decision to use bail-out stent. Case 2 had the appearance of a type B dissection, but the case was concerning due to a possible vessel threatening dissection as a result of a persistent dye hang-up in the dissection after contrast was cleared from the coronary tree.

## 4. Discussion

PCI using a DES is a safe treatment option for LMS disease in studies with long-term follow-up [29]. Whilst individual case reports involving LMS DCBs have been published [30,31,32], the SPARTAN-LMS study adds to the growing evidence that demonstrates the safety of DCB-only angioplasty for de novo LMS disease, compared with second-generation DES. After an average follow-up of 33.9 ± 19.9 months, there was no significant difference in all-cause or cardiovascular mortality both before and after propensity matching. True bifurcational distribution of LMS disease, peripheral vascular disease, COPD, AF, low eGFR, and femoral access was identified as an independent poor predictor of all-cause mortality. Lesion length, diabetes, prior CABG, and smoking were the only independent poor predictors of cardiovascular mortality.

The significant adverse factors associated with all-cause and cardiovascular mortalities in our study are consistent with various trials looking at mortality associated with coronary artery disease and PCI. Atrial fibrillation, renal failure, peripheral vascular disease, access site, and acute coronary syndromes are all factors which have been shown to have a significant effect on mortality after PCI [33,34,35,36,37,38,39].

All-cause and cardiovascular mortality in our study was higher compared to the PCI groups of NOBLE and EXCEL trials [2,40]. This reflects the fact that our study represents real-world data with inclusion of high-risk groups, such as STEMIs (7.8%), NSTEMIs (45.6%), and true bifurcation lesions (57.5%), with no age cut-off in to enter the registry. By contrast, both NOBLE and EXCEL trials recruited patients mainly with stable angina. In NOBLE, 82% of patients had stable angina, and STEMI patients were excluded all together [40]. Meanwhile, EXCEL was comprised 60.8% of patients with stable angina presentations and only 1.4% presented with STEMI [2]. Our outcomes are similar to those reported in the national British Cardiac Intervention Society (BCIS) audit data for LMS PCI [41]. Presentation with ACS was a significant predictor of mortality in our study, which could explain the higher mortality observed in our cohort. True bifurcation disease was not associated with all-cause or cardiovascular mortality in our study. However, previous studies have demonstrated that bifurcations themselves yield risks of cardiac mortality [4,5,33] and, in our study, there was a statistically significant usage of DCBs over DESs for true bifurcational disease to tackle this anatomical challenge [42,43,44].

DCB angioplasty practice has increasingly been utilised in our institution since 2011, with the first de novo LMS DCB treated in 2014. This reflects the gradual learning curve of a novel technique, starting with less complex lesions. In our centre, only operators performing DCB angioplasty in non-LMS lesions (more than 300 independent cases) have adopted a DCB-only approach for LMS PCI, and their initial cases were conducted with the support of another experienced DCB operator. Our practice focuses on optimal lesion preparation with a safe angioplasty result considered as the following: (1) the absence of a vessel threatening dissection (typically a type A or type B dissection without contrast hang-up); (2) TIMI 3 flow in the coronary vessels; (3) the absence of ≥30% recoil. This, combined with performing optimal balloon angioplasty, ensures adequate lesion preparation and denotes an appropriate lesion to treat with a DCB. This practice is based on our centre’s expertise, combined with previous DCB consensus documents, and has led to the development of the third consensus document [27]. This practice has been confirmed by our published safety data of immediate procedural complications [45], which showed acute/sub-acute vessel closure after discharge at 1%. Routinely, patients after elective PCI are discharged 6 h post-DCB and 4 h post-DES. A meta-analysis of acute vessel closure in DCB RCTs reported a rate of 2.6%, which was not significantly different to acute stent thrombosis [46]. Whilst early angioplasty reports highlighted an increased mortality when treating unprotected left main stem lesions compared with protected left main stem lesions with balloon angioplasty alone [47], combined with studies showing improved survival with CABG compared with medical therapy [48,49,50], the use of balloon angioplasty for left main stem disease was not commonly utilised. However, with increasing evidence now supporting the use of DESs [29], it was important to revisit the alternative of DCB angioplasty in treating LMS lesions.

Finally, the use of DCB angioplasty involves a shorter duration of dual antiplatelet therapy, with a one-month duration safe in elective angioplasty. This provides an additional benefit to favour DCB use in patients who are considered to be at a higher risk of bleeding.

### Limitations

This study is retrospective, non-randomised, and from a single centre; thus, it is associated with all the limitations and potential bias relevant to this type of study. However, our database was collated prospectively, and it gives an insight into the real-world treatment of LMS disease. Given the retrospective nature, there is a possibility that disease complexity was not matched between the two groups, and the possibility of selection bias exists. The DCB group was associated with significantly more bifurcation lesions and an older population. The propensity score matched analysis was performed in order to account for this. Furthermore, our institution is a large tertiary referral centre providing cardiac treatment to a population in excess of one million people with well-established high rates of DCB use. These results may not be generalisable to operators/centres with less experience in DCB angioplasty.

Our angiographic estimates of vessel diameter were based on visual assessment by the operator during the procedure and has not been quantified using QCA.

## 5. Conclusions

Whilst there is sufficient evidence for the use of DESs in LMS, there is a paucity of data exploring the role of DCBs in de novo LMS. This is the first study comparing DCB-only angioplasty for de novo LMS disease with second-generation DESs. It demonstrates the safety of DCB-only angioplasty in our own institution and suggests that a DCB-only strategy could be considered an alternative strategy to DESs in experienced centres, particularly for individuals that are at a high risk of prolonged dual antiplatelet therapy.

## Figures and Tables

**Table 1:** Baseline characteristics of the patient groups.

	Paclitaxel DCB (*n* = 41)	Second-Generation DES (*n* = 107)	*p*-Value
Age	Mean 73.8 ± 11.7	Mean 69.8 ± 11.1	0.048 *
Male	35 (85.4%)	82 (76.6%)	0.25
Weight (kg)	84.39 ± 19.10	80.81 ± 14.81	0.24
Hypercholesterolaemia	11 (26.8%)	26 (24.3%)	0.82
Hypertension	23 (56.1%)	56 (52.3%)	0.64
PVD	3 (7.3%)	5 (4.7%)	0.52
CVA	4 (9.8%)	6 (5.6%)	0.36
Prior CABG	1 (2.4%)	2 (1.9%)	0.82
Heart Failure	1 (2.4%)	3 (2.8%)	0.91
Previous PCI	8 (19.5%)	31 (29%)	0.21
Prior MI	9 (22%)	26 (24.3%)	0.70
eGFR	Mean 69.88 ± 21.87	Mean 73.43 ± 23.72	0.39
Smoking	24 (64.9%)	58 (56.3%)	0.39
COPD	3 (7.3%)	10 (9.3%)	0.71
Family History of IHD	4 (9.8%)	10 (9.3%)	0.93
Atrial Fibrillation	5 (12.2%)	4 (3.7)	0.052
Diabetes	13 (31.7%)	24 (22.4)	0.23
DAPT 1 month 12 months	19 (46.3%) 22 (53.7%)	4 (3.7%) 103 (96.3%)	<0.01
GPIIbIIIa use	14 (34.1%)	20 (18.7%)	0.06
ACS STEMI NSTEMI	5 (12.2%) 23 (56.1%)	10 (9.3%) 47 (43.9%)	0.37 Ɨ
ELECTIVE Stable Angina Staged	10 (24.4%) 3 (7.3%)	43 (40.2.9) 7 (6.5%)	

Baseline patient characteristics of patients treated with DCB or DES for de novo LMS. * denotes statistical significance in its composition within the DCB group. Ɨ denotes a *p* value of a composite of STEMI and NSTEMI vs. angina and staged groups. DAPT: dual antiplatelet therapy; GPIIbIIIa: Glycoprotein IIBIIIa inhibitor.

**Table 2:** Procedural characteristics of both patient groups.

	Paclitaxel DCB (*n* = 41)	Second-Generation DES (*n* = 107)	*p*-Value
Radial access	35 (85.4%)	88 (82.2%)	0.67
Vessel diameter (mm)	3.84 ± 0.26	4.33 ± 0.60	<0.001 *
Lesion length (mm)	21.71 ± 7.80	24.72 ± 14.32	0.20
True bifurcation	30 (73.2%)	52 (48.6%)	0.006 *
Contrast	144.5 ± 41.3	176.5 ± 67.1	0.006 *
Intravascular imaging (OCT or IVUS)	14 (35.9%)	82 (76.6%)	<0.001
Coronary dissection after DCB - Type A - Type B	4 (9.8%) 16 (39.0%)	N/A	
Calcium modification			
Rotational atherectomy	1 (2.4%)	7 (6.5%)	0.32
Shockwave lithotripsy	0 (0%)	1 (0.9%)	0.53
Cutting balloon	1 (2.4%)	0 (0%)	0.61

Angiographic characteristics of patients treated with DCB or DES for de novo disease. * denotes statistical significance in its comparison between the DCB and DES groups.

## Data Availability

Data is not publically available due to data protection governance and in keeping with data handling policy from our ethics application.
